# Biochemical and Endocrine Parameters for the Discrimination and Calibration of Bipolar Disorder or Major Depressive Disorder

**DOI:** 10.3389/fpsyt.2022.875141

**Published:** 2022-06-20

**Authors:** Yuncheng Zhu, Haifeng Ji, Zhiang Niu, Hongmei Liu, Xiaohui Wu, Lu Yang, Zuowei Wang, Jun Chen, Yiru Fang

**Affiliations:** ^1^Division of Mood Disorders, Shanghai Hongkou Mental Health Center, Shanghai, China; ^2^Clinical Research Center and Division of Mood Disorders, Shanghai Mental Health Center, Shanghai Jiao Tong University School of Medicine, Shanghai, China; ^3^Division of Psychiatry, Shanghai Changning Mental Health Center, Shanghai, China; ^4^CAS Center for Excellence in Brain Science and Intelligence Technology, Shanghai, China; ^5^Shanghai Key Laboratory of Psychotic Disorders, Shanghai, China

**Keywords:** bipolar disorder, major depressive disorder, mood disorders, gender, biological markers

## Abstract

**Objectives:**

Conventional biochemical indexes may have predictive values in clinical identification between bipolar disorder (BD) and major depressive disorder (MDD).

**Methods:**

This study included 2,470 (BD/MDD = 1,333/1,137) hospitalized patients in Shanghai as training sets and 2,143 (BD/MDD = 955/1,188) in Hangzhou as test sets. A total of 35 clinical biochemical indexes were tested, including blood cells, immuno-inflammatory factors, liver enzymes, glycemic and lipid parameters, and thyroid and gonadal hormones. A stepwise analysis of a multivariable logistic regression was performed to build a predictive model to identify BD and MDD.

**Results:**

Most of these biochemical indexes showed significant differences between BD and MDD groups, such as white blood cell (WBC) in the hematopoietic system, uric acid (UA) in immuno-inflammatory factors, direct bilirubin (DBIL) in liver function, lactic dehydrogenase (LDH) in enzymes, and fasting blood glucose (FBG) and low-density lipoprotein (LDL) in glucolipid metabolism (*p*-values < 0.05). With these predictors for discrimination, we observed the area under the curve (AUC) of the predictive model to distinguish between BD and MDD to be 0.772 among men and 0.793 among women, with the largest AUC of 0.848 in the luteal phase of women. The χ^2^ values of internal and external validation for male and female datasets were 2.651/10.264 and 10.873/6.822 (*p*-values < 0.05), respectively. The AUCs of the test sets were 0.696 for males and 0.707 for females.

**Conclusion:**

Discrimination and calibration were satisfactory, with fair-to-good diagnostic accuracy and external calibration capability in the final prediction models. Female patients may have a higher differentiability with a conventional biochemical index than male patients.

**Trial Registration:**

ICTRP NCT03949218. Registered on 20 November 2018. Retrospectively registered. https://www.clinicaltrials.gov/ct2/show/NCT03949218?id=NCT03949218&rank=1.

## Introduction

The National Depressive and Manic-Depressive Association investigated the diagnosis and treatment conditions of bipolar disorder (BD) in 2000, and the results indicated that 69% of cases were misdiagnosed (1). A mean of four psychiatrists were consulted by a patient with BD before receiving the accurate diagnosis, with over one-third taking 10 years before being correctly diagnosed (1). The proportion of BD misdiagnosed as major depressive disorder (MDD) in clinical practice was reported as 20.8% nationwide in China (2). The long-term consistency rate of MDD cases across a 10-year study was only 45.5% (3) compared with 71.9% in a 7-year cohort study of BD cases (4). The misdiagnosis rate has not substantially decreased over the past nearly 20 years.

Mitochondrial malfunction, inflammatory cytokines, and activated microglia were pointed out as potential biomarkers of BD from the perspective of oxidative stress and neurogenic inflammation (5–7). The oxidative stress of mood disorders may lead to inflammatory changes in multisystem function, which may be detected by laboratory examination (8, 9). Downstream parameters of inflammatory response are likely to be more available for clinical use in routine blood testing using peripheral blood (10).

After that thought, we extracted our database and found 14 inflammatory markers of patients with mood disorders having different elevated levels (11), such as uric acid (UA), direct bilirubin (DBIL), and lactic dehydrogenase (LDH). The data-driven analytics based on inflammation have not yet been proven between BD and MDD. Thus, we speculated that biochemical and endocrine parameters deserve potential development (12).

To help medical professionals, discovering an informative diagnostic tool to improve the uniformity of clinical judgment, we have created new parametric prediction models for discriminating between BD and MDD based on conventional biochemical indexes and hormones. Due to the different range of biochemical parameters between sexes, sex difference is a strong heterogeneous factor that needs to be fully considered (13). Therefore, a male model and a female model at stage 1 and three submodels for different phases of the menstrual cycle of female patients at stage 2 were built to make the precise analysis. In this study, the effectiveness of our model is discussed.

## Patients and Methods

### Trial Design

This study was a retrospective, cross-sectional, and real-world study. We registered as a clinical trial (No. NCT03949218) at the International Clinical Trials Registry Platform and obtained ethical approval 2019-15R from the Shanghai Mental Health Center (SMHC). Informed consent restricts the use of the material to scientific research purposes only, according to the Declaration of Helsinki, and permission to take informed consent is formally waived by the approving committee.

All data were gathered by the Information Department of SMHC and Hangzhou Seventh People's Hospital (HSPH). Information engineers developed the Hospital Information System (HIS) for searching of clinical big data based on the code of ICD-10 criteria, laboratory database, medical examination database, and drug database (Supplementary Material 1).

The patients' personal identifiable information was redacted to protect patient privacy and identity before being provided for analysis.

### Participants

The population-based sample used for this report included 4,647 hospitalized mood disorder patients from January 2009 to December 2018 in SMHC and 3,029 from January 2017 to December 2019 in HSPH. The mental examination was conducted by a three-level ward round, including at least one chief physician. We extracted the diagnostic code of ICD-10 from the discharge abstract of each patient. The biochemical data and electronic medical records from the HIS are available. Predictors considered were age, age at onset, disease duration, sex and clinical biochemical data at admission regarding the hematopoietic system, immuno-inflammatory indexes, liver function, glucolipidmetabolism, thyroid function, and sex hormones (11). The 35 indexes and their normal ranges are given in Supplementary Material 2.

We collected fasting venous blood between 6:00 and 8:00 a.m. using a set of standard operating procedures at the nurses' workstation. The inpatients had neither tobacco use nor alcohol consumption at least 12 h before the blood specimen collection. An electrochemical luminescence immunoassay (ECLIA) was performed using the Roche Cobas e601 automatic electrochemiluminescence immunoassay system (14), provided by SMHC and HSPH. After the blood test, clinical medication and dosage adjustments would be arranged by a doctor-in-charge starting at 8 a.m. each day.

Inclusion criteria were as follows: 1. diagnosed BD, MDD, or their subtypes according to the ICD-10; 2. available biochemical data in the HIS; and 3. hospitalized patients with no substance-related or addictive disorders. Exclusion criteria were as follows: 1. comorbidity with other mental disorders; 2. pregnancy or postpartum lactation; 3. severe physical illness; and 4. indefinitive menstrual history for female patients.

**Figure 1:**
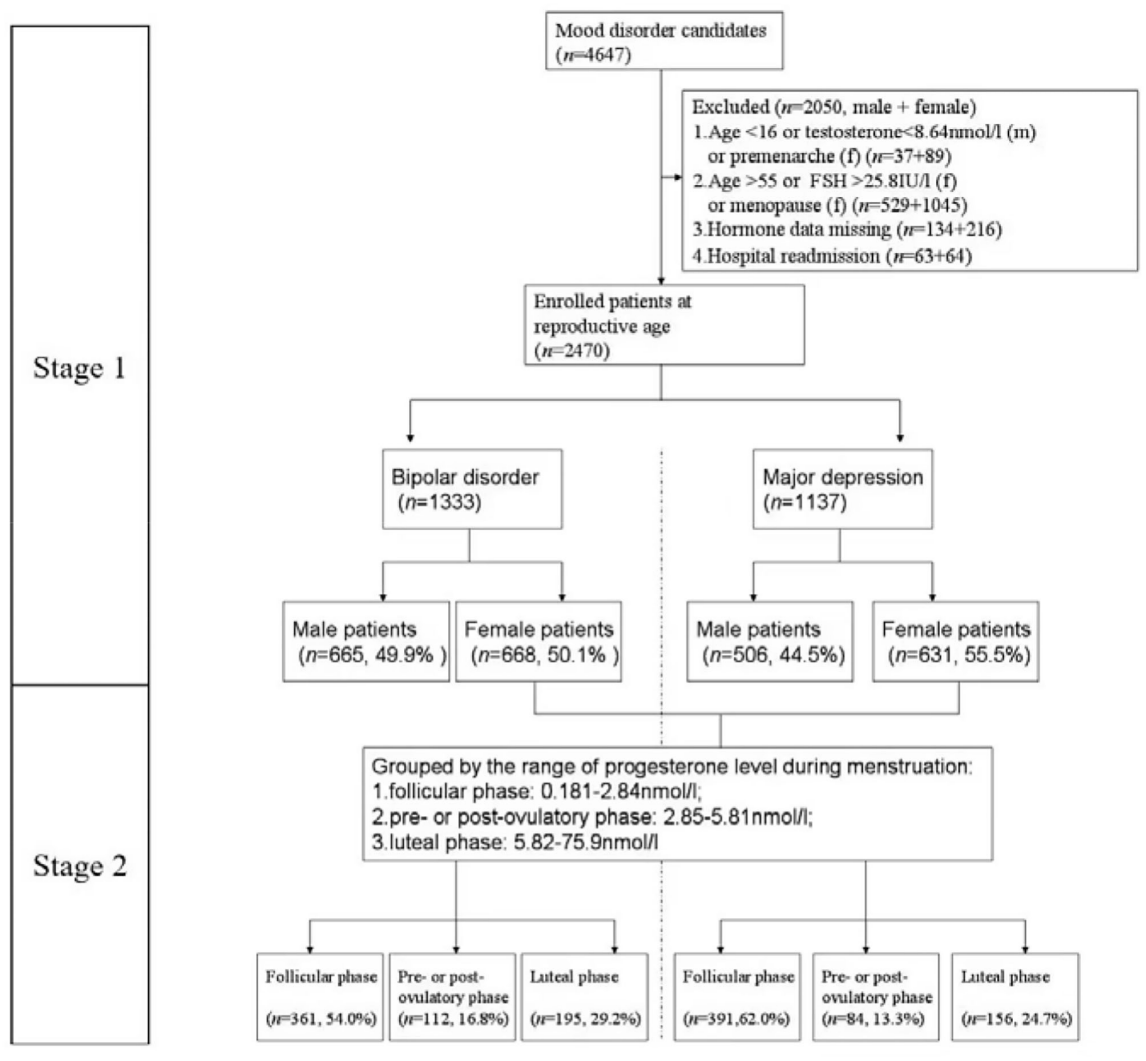
Flowchart of screening process and data classification.

For this study, we continued to screen the database and excluded the following patients to focus on patients of reproductive age: 1. Age <16 or testosterone of male <8.64 nmol/L or pre-menarche female; 2. age > 55 or FSH of female > 25.8 IU/L or menopause female; 3. hormone data missing; 4. hospital readmission. With the exclusion of the above cases, a total of 2,470 patients were selected (BD = 1,333, MDD = 1,137) as training data sets. The median observation period was 5 years and it was taken to convert the diagnosis of BD by 27.3 ± 22.4 months from the first hospitalization of the BD-converters. We actually identified 64 BD-converters from patients with MDD, who were counted in the BD group. Among the 3,029 patients in HSPH, we excluded 886 patients [(age <16 or testosterone of male <8.64 nmol/L or pre-menarche female, *n* = 406) and (age > 55 or menopause female, *n* = 480)]. Finally, there were 2,143 patients matched (BD = 955, MDD = 1,188) as testing datasets. To ensure the accuracy and reliability of the grouping by sex steroids, we randomly selected 1% of the cases from female patients to check their menstrual histories and confirmed that the results were consistent with what would be expected given the patients' menstrual phase. We divided our research into two stages (refer to Figure 1 for a flow diagram of sample selection) (11).

### Outcome

Many indexes need to be discussed separately for different normal ranges due to gender differences. Therefore, the two diseases were classified into gender subgroups if necessary.

Outcomes of interest were 35 indexes in five domains: hematopoietic system (WBC, neutrophil, RBC, hemoglobin, and platelet); immuno-inflammatory indexes (ESR, CRP, UA, and prealbumin); liver function (albumin, globulin, TBIL, DBIL, IDBIL, GPT, GOT, LDH, GGT, and ALP); glucolipid metabolism (FBG, TCH, triglyceride, HDL, and LDL); hypothalamic-pituitary-thyroid (HPT) axis; and sex steroids (TSH, TT4, FT4, TT3, FT3, testosterone, prolactin, LH, FSH, estradiol, and progesterone). The levels of sex steroids (i.e., LH, FSH, estradiol, and progesterone) fluctuating with the menstrual cycle in females were not compared between female subgroups.

### Predictors

In the session of correlation analysis, we considered these variables that may yield potential collinearity to avoid affecting the final results. Before building the regression model, the parameters were set to be excluded, provided that the potential multicollinearity was above five of the variance inflation factor. Based on the principle, we removed neutrophil, CRP, hemoglobin, TBIL, GOT, TCH, albumin, globulin, TT4 and TT3. The results showed no multicollinearity between the 25 remaining independent variables of the gender subgroups in Supplementary Material 3.

Then, the remaining data were extracted for each patient. In the case of repeated tests, the first report of blood examination of each patient during hospitalization was used.

### Missing Data

To ensure reliability of the data, we excluded patients (*n* = 350) who had plenty of data loss (≥30% estimated data). In this study, we continued to screen the database and excluded patients with hormone data missing. The hormone data must include TSH, FT3, FT4, FSH, testosterone in men, and progestin in women. Patients were excluded from the development of models if they had no information on any of the prediction parameters.

### Statistical Analysis

Statistical analyses were performed using SPSS version 22.0. Comparisons of the candidate biochemical indices between BD and MDD were performed *via* Student's *t-*test (normally distributed data) or rank sum test (skewed distribution data), as appropriate. All tests were two-tailed, and statistical significance was defined as a *p-*value <0.05. The effect sizes were employed by the *Cohen's d* or *OR*. For identification of the biochemical index between BD and MDD, a forward conditional selection of multivariable logistic regression was performed for training sets, with *p-*value criteria of 0.05 for entry and 0.10 for removal of variables. Hierarchical multiple regression was performed for the test sets using the same parameters. The diagnostic categorical variables were listed as 1 = BD and 2 = MDD. We removed the variables that could cause potential multicollinearity before constructing a conditional forward pattern of the model. The chosen predictive factors were based on previous high-quality studies, reviews, and meta-analyses (15–33). Then, a receiver operating characteristic (ROC) curve was plotted *via* the probabilities of the predicted values to determine the predictable validity of the logistic regression model. The capability of the prediction models was calibrated *via* Hosmer–Lemeshow goodness-of-fit test. The AUCs and standard errors of the ROC curve were entered into MedCalc version 19.6.1 *z* test to compare the independent samples.

## Results

### Comparison Between BD and MDD Groups

We analyzed 1,333 patients (49.9% male, *n* = 665) with BD and 1,137 with MDD (44.5% male, *n* = 506). Table 1 presents the comparison between the groups. There were no differences neither in RBC, hemoglobin, ESR, albumin, globulin, TBIL, GGT, ALP, TCH, HDL, LDL, TT4, FSH, or progesterone levels between the male subgroups nor in TBIL, GGT, ALP, TCH, HDL, TSH, or TT4 between the female subgroups (*p*-values > 0.05), while the rest of the results showed significantly differences between gender subgroups (*p*-values < 0.05).

**Table 1:** Comparison between BD and MDD groups in the gender subgroup.

**Variables (*n*, BD/MDD)**	**BD**	**MDD**	***t/Z***	***p***	**Cohen's *d***
Age (y)	29.71 ± 9.78	32.68 ± 11.29	6.105	<0.001	0.247
Male (665/506)	30.29 ± 10.44	34.03 ± 11.67	5.263	<0.001	0.311
Female (668/631)	29.13 ± 9.06	31.60 ± 10.87	3.656	<0.001	0.203
Disease duration (y)	6.50 ± 6.68	5.47 ± 6.48	4.295	<0.001	0.173
Male (665/506)	6.80 ± 7.16	5.25 ± 6.41	3.441	0.001	0.203
Female (668/631)	6.19 ± 6.13	5.57 ± 6.23	2.300	0.021	0.128
Age at onset (y)	23.21 ± 8.78	27.21 ± 10.83	10.460	<0.001	0.422
Male (665/506)	23.49 ± 9.11	28.78 ± 11.01	4.694	<0.001	0.277
Female (668/631)	22.94 ± 8.42	26.03 ± 10.25	7.988	<0.001	0.443
Hematopoietic system
WBC (10^9^/L)	7.83 ± 2.53	6.95 ± 2.25	9.421	<0.001	/
Male (665/506)	7.98 ± 2.51	7.16 ± 2.33	6.099	<0.001	0.360
Female (668/631)	7.68 ± 2.53	6.77 ± 2.17	6.865	<0.001	0.381
Neutrophil (10^9^/L)	4.95 ± 2.31	4.30 ± 2.08	7.959	<0.001	/
Male (665/506)	5.06 ± 2.27	4.41 ± 2.16	5.798	<0.001	0.342
Female (668/631)	4.84 ± 2.34	4.21 ± 2.01	5.213	<0.001	0.289
RBC (10^12^/L)	4.74 ± 0.54	4.65 ± 0.57	3.982	<0.001	/
Male (665/506)	5.07 ± 0.46	5.01 ± 0.49	1.896	0.058	/
Female (668/631)	4.41 ± 0.40	4.36 ± 0.45	2.320	0.021	0.129
Hemoglobin (g/L)	140.66 ± 16.61	138.74 ± 17.36	2.745	0.006	/
Male (665/506)	151.93 ± 11.52	151.83 ± 12.85	0.583	0.560	/
Female (668/631)	129.28 ± 12.67	128.15 ± 12.37	2.180	0.029	0.121
Platelet (10^9^/L)	249.51 ± 66.56	235.65 ± 64.93	5.674	<0.001	/
Male (665/506)	235.23 ± 56.86	223.82 ± 58.61	3.357	0.001	0.198
Female (668/631)	263.73 ± 72.27	245.14 ± 68.17	4.772	<0.001	0.265
Immunoinflammatory indexes
ESR (mm/h)	7.77 ± 6.66	7.11 ± 6.20	2.515	0.012	/
Male (657/484)	4.61 ± 4.68	4.24 ± 4.43	1.331	0.183	/
Female (661/603)	10.87 ± 6.85	9.42 ± 6.45	4.010	<0.001	0.226
CRP (mg/L)	2.86 ± 5.63	2.02 ± 3.45	4.096	<0.001	/
Male (561/377)	3.07 ± 6.23	2.29 ± 4.16	2.300	0.022	0.153
Female (588/463)	2.65 ± 4.99	1.80 ± 2.74	3.530	<0.001	0.219
UA (μmol/L)	351.4 ± 106.87	311.90 ± 93.58	9.181	<0.001	/
Male (580/451)	397.70 ± 103.09	359.44 ± 92.57	6.269	<0.001	0.394
Female (593/564)	306.21 ± 89.84	273.89 ± 75.26	6.470	<0.001	0.381
Prealbumin (mg/L)	264.02 ± 100.51	281.45 ± 76.69	5.788	<0.001	/
Male (584/454)	289.41 ± 68.61	313.46 ± 79.42	4.805	<0.001	0.301
Female (600/569)	239.30 ± 118.86	255.91 ± 63.86	5.404	<0.001	0.316
Liver function
Albumin (g/L)	42.83 ± 3.87	42.34 ± 3.96	2.922	0.004	/
Male (586/454)	43.76 ± 3.64	43.59 ± 3.64	0.717	0.473	/
Female (600/570)	41.93 ± 3.88	41.35 ± 3.93	2.539	0.011	0.149
Globulin (g/L)	27.61 ± 4.19	27.19 ± 3.75	2.028	0.043	/
Male (586/454)	26.85 ± 3.75	26.52 ± 3.54	1.446	0.149	/
Female (600/570)	28.34 ± 4.46	27.72 ± 3.83	2.539	0.011	0.149
TBIL (μmol/L)	15.25 ± 7.43	14.74 ± 7.22	2.040	0.041	/
Male (585/455)	16.78 ± 7.98	16.53 ± 7.68	0.678	0.498	/
Female (600/570)	13.75 ± 6.51	13.31 ± 6.49	1.530	0.126	/
DBIL (μmol/L)	2.02 ± 1.38	3.10 ± 2.17	12.810	<0.001	/
Male (586/454)	2.28 ± 1.55	3.49 ± 2.39	9.138	<0.001	0.571
Female (640/587)	1.76 ± 1.12	2.77 ± 1.88	9.880	<0.001	0.565
IDBIL (μmol/L)	13.23 ± 6.68	11.64 ± 6.50	6.751	<0.001	/
Male (585/454)	14.50 ± 7.16	13.02 ± 7.04	3.825	<0.001	0.239
Female (600/570)	11.99 ± 5.92	10.53 ± 5.81	5.222	<0.001	0.306
GPT (U/L)	25.69 ± 23.05	23.58 ± 24.67	4.651	<0.001	/
Male (585/454)	31.87 ± 28.11	28.92 ± 27.05	3.182	0.001	0.199
Female (600/570)	19.65 ± 14.36	19.33 ± 21.70	2.798	0.005	0.164
GOT (U/L)	24.82 ± 18.15	21.04 ± 14.18	8.036	<0.001	/
Male (587/454)	28.42 ± 23.13	22.32 ± 12.84	6.409	<0.001	0.401
Female (603/571)	21.32 ± 10.25	20.02 ± 15.10	4.617	<0.001	0.270
LDH (U/L)	152.36 ± 59.41	130.86 ± 31.49	11.209	<0.001	/
Male (587/453)	155.98 ± 70.04	132.28 ± 28.35	6.882	<0.001	0.430
Female (603/571)	148.83 ± 46.59	129.73 ± 33.75	8.794	<0.001	0.514
GGT (U/L)	21.24 ± 24.56	21.64 ± 30.35	1.103	0.270	/
Male (586/454)	27.17 ± 31.69	28.36 ± 41.40	0.585	0.558	/
Female (600/570)	15.44 ± 12.02	16.29 ± 15.07	0.196	0.844	/
ALP (U/L)	61.07 ± 19.20	61.44 ± 21.31	0.231	0.817	/
Male (586/454)	65.95 ± 20.49	67.93 ± 23.60	1.288	0.198	/
Female (600/570)	56.30 ± 16.53	56.26 ± 17.69	0.494	0.622	/
Glucolipid metabolism
FBG (mmol/L)	5.78 ± 1.58	5.43 ± 1.42	7.580	<0.001	/
Male (612/457)	5.85 ± 1.51	5.63 ± 1.73	3.895	<0.001	0.241
Female(612/581)	5.71 ± 1.64	5.28 ± 1.10	6.513	<0.001	0.377
TCH (mmol/L)	4.45 ± 0.98	4.54 ± 1.02	1.600	0.110	/
Male (539/423)	4.44 ± 0.96	4.49 ± 0.97	0.494	0.621	/
Female (544/528)	4.46 ± 1.00	4.58 ± 1.06	1.643	0.100	/
Triglyceride (mmol/L)	1.21 ± 0.98	1.30 ± 0.98	3.596	<0.001	/
Male (539/423)	1.43 ± 1.17	1.56 ± 1.18	2.111	0.035	0.137
Female (543/528)	0.99 ± 0.69	1.11 ± 0.72	4.229	<0.001	0.259
HDL (mmol/L)	1.31 ± 0.33	1.33 ± 0.35	0.813	0.416	/
Male (539/423)	1.20 ± 0.28	1.23 ± 0.30	1.167	0.243	/
Female (544/528)	1.42 ± 0.33	1.41 ± 0.37	1.184	0.236	/
LDL (mmol/L)	2.49 ± 0.85	2.59 ± 0.88	2.372	0.018	/
Male (539/423)	2.56 ± 0.86	2.58 ± 0.81	0.398	0.690	/
Female (544/528)	2.43 ± 0.84	2.59 ± 0.92	3.046	0.002	0.186
HPT axis
TSH (mIU/L)	2.71 ± 3.27	2.38 ± 2.90	1.660	0.097	/
Male (664/504)	2.50 ± 1.96	2.30 ± 3.66	2.979	0.003	0.176
Female (666/628)	2.91 ± 4.17	2.45 ± 2.09	0.052	0.958	/
TT4 (nmol/L)	94.25 ± 24.41	91.59 ± 25.46	0.158	0.875	/
Male (665/505)	92.31 ± 22.17	91.71 ± 24.30	1.641	0.101	/
Female (665/629)	96.18 ± 26.35	91.49 ± 26.37	1.480	0.139	/
FT4 (pmol/L)	17.56 ± 5.76	15.76 ± 4.37	8.376	<0.001	/
Male (665/506)	17.67 ± 4.88	16.13 ± 4.25	4.793	<0.001	0.283
Female (667/628)	17.46 ± 6.52	15.47 ± 4.44	6.398	<0.001	0.356
TT3 (nmol/L)	1.60 ± 0.42	1.52 ± 0.33	5.838	<0.001	/
Male (662/506)	1.65 ± 0.35	1.57 ± 0.32	3.664	<0.001	0.216
Female (665/628)	1.56 ± 0.47	1.48 ± 0.33	3.172	0.002	0.177
FT3 (pmol/L)	4.76 ± 1.48	4.28 ± 1.08	11.914	<0.001	/
Male (663/503)	4.95 ± 0.87	4.53 ± 1.04	8.055	<0.001	0.476
Female (667/629)	4.57 ± 1.88	4.07 ± 1.06	7.618	<0.001	0.423
HPG axis
Testosterone (nmol/L)
Male (646/489)	15.50 ± 7.42	13.64 ± 7.27	4.048	<0.001	0.243
Female (666/630)	1.27 ± 0.77	1.04 ± 0.60	6.510	<0.001	0.362
Prolactin (mIU/L)
Male (659/482)	599.17 ± 511.28	441.87 ± 443.01	6.958	<0.001	0.417
Female (662/615)	1151.2 ± 1173.9	804.3 ± 1069.0	8.459	<0.001	0.477
LH (IU/L)
Male (403/315)	5.67 ± 2.64	5.25 ± 2.51	2.247	0.025	0.169
FSH (IU/L)
Male (403/315)	4.50 ± 2.72	4.82 ± 3.09	0.985	0.325	/
Estradiol (pmol/L)
Male (646/486)	109.43 ± 61.72	83.72 ± 47.78	7.330	<0.001	0.440
Progesterone (nmol/L)
Male (403/315)	1.69 ± 1.18	1.59 ± 1.10	1.069	0.285	/

*BD, bipolar disorder; MDD, major depressive disorder; HPT, hypothalamic-pituitary-thyroid; HPG, hypothalamic-pituitary-gonad; WBC, white blood cell; ESR, erythrocyte sedimentation rate; CRP, C-reactive protein; RBC, red blood cell; UA, uric acid; TBIL, total bilirubin; DBIL, direct bilirubin; IDBIL, indirect bilirubin; GPT, glutamic-pyruvic transaminase; GOT, glutamic-oxalacetic transaminase; LDH, lactic dehydrogenase; GGT, glutamyltranspeptidase; ALP, alkaline phosphatase; FBG, fasting blood glucose; TCH, total cholesterol; HDL, high-density lipoprotein; LDL, low-density lipoprotein; TSH, thyroid-stimulating hormone; TT4, total thyroxine; FT4, free thyroxine; TT3, total triiodothyronine; FT3, free triiodothyronine; LH, luteinizing hormone; FSH, follicle-stimulating hormone*.

### Regression Analysis of Distinguishing BD From MDD Patients by Biochemical Indexes

We tried to further associate BD and MDD with the potential risk factors, including biochemical indexes and thyroid hormones. We analyzed the subgroup of male patients first and then female patients. There were 954 male (536 vs. 418) and 1,060 female (540 vs. 520) patients who were included in the first step analysis; 217 and 239 cases missing at least one index were excluded, respectively. The regression models were finally constructed, while the other variables did not enter the models (Table 2).

**Table 2:** Regression analysis for diagnostic predictors to BD vs. MDD.

	**β**	**S.E**	***P***	**OR (95% CI)**
**Male model**
Constant	2.351	0.682	0.001	10.500
DBIL	0.388	0.051	<0.001	1.474 (1.334, 1.630)
LDH	−0.013	0.002	<0.001	0.987 (0.982, 0.992)
UA	−0.003	0.001	0.002	0.997 (0.995, 0.999)
TSH	−0.090	0.039	0.021	0.914 (0.846, 0.986)
FT3	−0.389	0.094	<0.001	0.678 (0.564, 0.815)
IDBIL	−0.041	0.012	0.001	0.960 (0.937, 0.983)
Prealbumin	0.004	0.001	0.001	1.004 (1.002, 1.006)
HDL	0.601	0.274	0.029	1.823 (1.065, 3.121)
**Female model**
Constant	3.789	0.621	<0.001	44.210
DBIL	0.608	0.063	<0.001	1.836 (1.622, 2.079)
LDH	−0.010	0.002	<0.001	0.990 (0.985, 0.994)
UA	−0.006	0.001	<0.001	0.995 (0.993, 0.996)
TSH	−0.094	0.037	0.010	0.910 (0.847, 0.978)
FT3	−0.238	0.081	0.026	0.788 (0.672, 0.924)
IDBIL	−0.036	0.014	0.009	0.965 (0.939, 0.991)
WBC	−0.111	0.035	0.002	0.895 (0.835, 0.958)
LDL	0.290	0.086	0.001	1.337 (1.128, 1.583)
FBG	−0.186	0.065	0.004	0.830 (0.730, 0.943)
GGT	0.017	0.006	0.005	1.017 (1.005, 1.030)
Triglyceride	0.319	0.120	0.008	1.375 (1.088, 1.739)

*DBIL, direct bilirubin; LDH, lactic dehydrogenase; UA, uric acid; TSH, thyroid-stimulating hormone; FT3, free triiodothyronine; IDBIL, indirect bilirubin; HDL, high-density lipoprotein; WBC, white blood cell; LDL, low-density lipoprotein; FBG, fasting blood glucose; GGT, glutamyltranspeptidase*.

**Figure 2:**
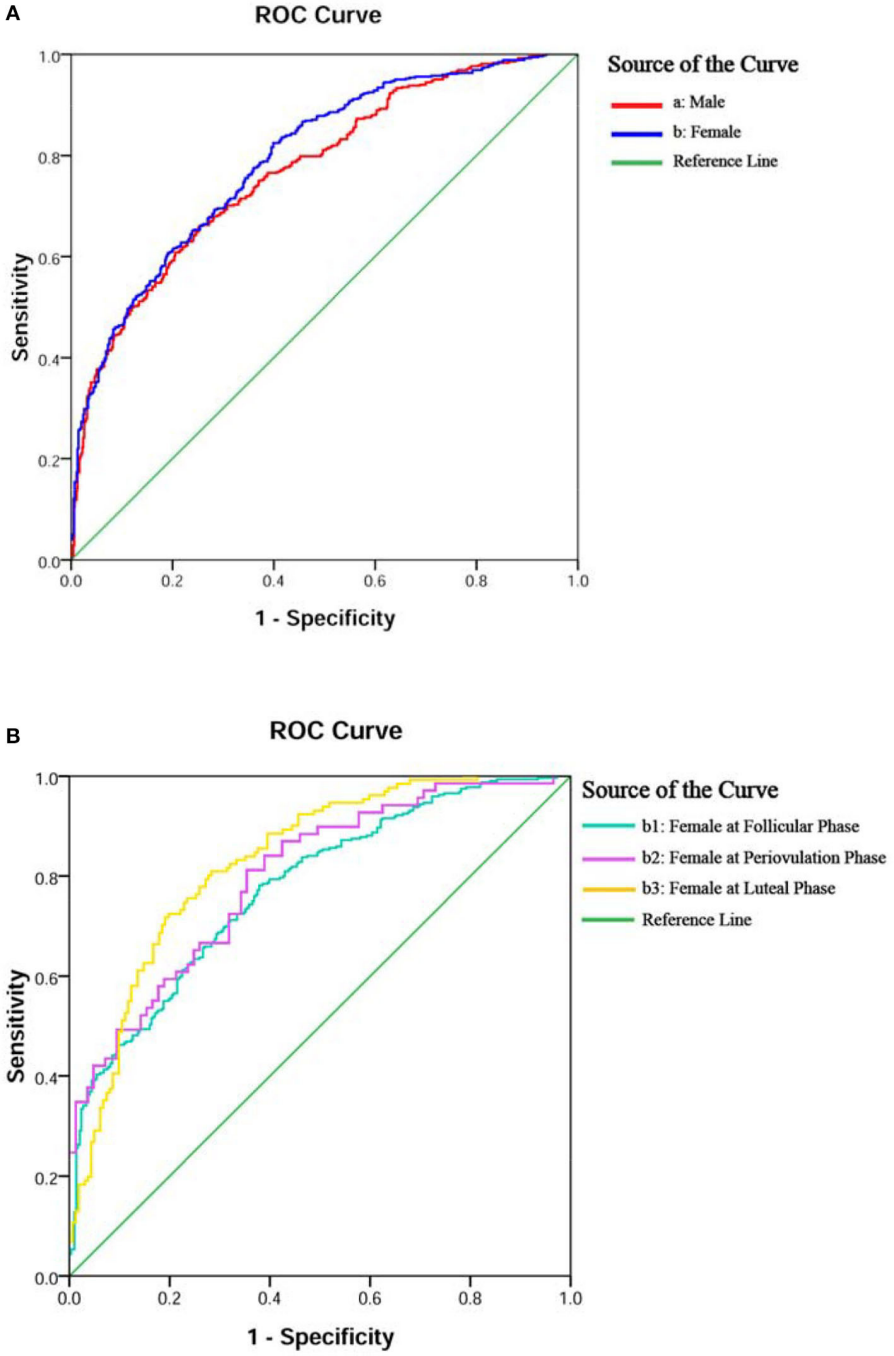
ROC curves of the logistic regression model for diagnostic accuracy of BD or MDD. Diagnostic accuracies of the models were yielded by gender in **(A,B)** and female subgroups in (**B**,b1–3).

The ROC curve in Figures 2A,B showed fair diagnostic accuracies of the models by gender, where the area under the curve (AUC) of the ROC were 0.772 (95% CI: 0.743–0.802) in males and 0.793 (95% CI: 0.767–0.820) in females, and the best cutoff values (Youden index) were 0.406 and 0.426, as shown in Table 3. We also obtained two balanced algorithm accuracies of 70.3% in males and 71.3% in females. There was no difference in the AUC between genders (*z* = 1.058, *p* = 0.290).

**Table 3:** Discrimination and calibration of the algorithm performance of biochemical index and HPT axis by BD/MDD diagnoses.

	**Youden index**	**AUC**	**95% CI for AUC**		**Calibration** ^**a**^	
			**Lower**	**Upper**	***χ2***	***p***
Male	0.406	0.772	0.743	0.802	2.651	0.954
Female	0.426	0.793	0.767	0.820	10.264	0.247
**Female subgroups**
Follicle	0.413	0.777	0.741	0.813	9.461	0.305
Periovulation	0.482	0.788	0.717	0.860	11.840	0.159
Luteum	0.571	0.848	0.805	0.891	5.875	0.661

*^a^Hosmer–Lemeshow goodness-of-fit test. AUC, the area under the curve*.

### Regression Analysis of Distinguishing Female BD From MDD Patients by Biochemical Indexes Subgrouped by Different Menstrual Cycles (Follicular, Periovulatory, and Luteal Phase)

In addition, we grouped female patients by their three physiological periods, as shown in Figure 1 (stage 2). There were 613 (293 vs. 320), 154 (85 vs. 69), and 293 (162 vs. 131) female patients who were included in the analysis, respectively, and the rest of the cases (139, 42, and 58) were excluded, respectively, for missing at least one of the indexes. Three regression models were constructed, as shown in Table 4.

**Table 4:** Regression analysis for female diagnostic predictors to BD vs. MDD.

	**β**	**S.E**	***p***	**OR (95% CI)**
				
**Follicular model**
Constant	2.258	0.700	0.001	9.564
DBIL	0.600	0.079	<0.001	1.822 (1.561, 2.128)
LDH	−0.011	0.003	<0.001	0.989 (0.984, 0.995)
UA	−0.005	0.001	<0.001	0.995 (0.993, 0.998)
FT3	−0.326	0.107	0.002	0.721 (0.585, 0.889)
WBC	−0.128	0.045	0.004	0.880 (0.805, 0.960)
LDL	0.354	0.111	0.001	1.425 (1.146, 1.772)
ALP	0.016	0.006	0.004	1.016 (1.005, 1.027)
**Periovulatory model**
Constant	−0.123	0.691	0.032	0.884
DBIL	0.710	0.156	<0.001	2.034 (1.498, 2.761)
LDH	−0.020	0.006	0.001	0.980 (0.969, 0.991)
GPT	0.053	0.019	0.005	1.055 (1.016, 1.095)
**Luteal model**
Constant	7.155	1.299	<0.001	1,281
DBIL	0.562	0.121	<0.001	1.755 (1.383, 2.226)
LDH	−0.022	0.006	<0.001	0.978 (0.967, 0.990)
UA	−0.009	0.002	<0.001	0.991 (0.987, 0.996)
TSH	−0.192	0.086	0.026	0.825 (0.697, 0.977)
IDBIL	−0.075	0.027	0.005	0.927 (0.879, 0.978)
WBC	−0.234	0.078	0.003	0.792 (0.679, 0.923)
LDL	0.481	0.178	0.007	1.617 (1.141, 2.292)
FBG	−0.347	0.134	0.010	0.707 (0.544, 0.919)
GGT	0.031	0.012	0.010	1.031 (1.007, 1.055)

*DBIL, direct bilirubin; LDH, lactic dehydrogenase; UA, uric acid; TSH, thyroid-stimulating hormone; FT3, free triiodothyronine; WBC, white blood cell; LDL, low-density lipoprotein; ALP, alkaline phosphatase; GPT, glutamic-pyruvic transaminase; IDBIL, indirect bilirubin; FBG, fasting blood glucose; GGT, glutamyltranspeptidase*.

The ROC curves in Figure 2B, b1–3 displayed the good diagnostic accuracy of these models, where the AUCs of the ROC were 0.777–0.848 and the Youden indexes were 0.413–0.571. Internal validation on the training sets, the results of the chi-square value (χ^2^) of the Hosmer–Lemeshow test were 2.651 for males, 10.264 for females, and 5.875–11.840 for females with different menstrual cycles. There were no differences in the AUC between the follicular phase and the periovulatory phase (*z* = 0.273, *p* = 0.785) nor between the periovulatory phase and the luteal phase (*z* = 1.422, *p* = 0.155). However, the difference between the periovulatory phase and the luteal phase was statistically significant (*z* = 2.498, *p* = 0.013).

The χ^2^ values of internal and external validation for male and female datasets were 2.651/10.264 and 10.873/6.822 (*p*-values < 0.05), respectively. The AUCs of the test sets were 0.696 (95% CI: 0.659–0.732) for males and 0.707 (95% CI: 0.678–0.735) for females. The differences in the AUC between training sets and test sets of gender subgroups were statistically significant (males, *z* = 3.244, *p* = 0.001; females, *z* = 3.839, *p* = 0.001). There were no statistical differences between the expected and the observed values for the five models (*p*-values > 0.05). The predictive models showed good calibration capability, as shown in Table 4.

Finally, we summarized the significant positive or negative correlation coefficients (*p*-values < 0.05) in those regression models for qualitative analysis. These predictors yielded completely consistent performance if more than one predictor was marked among the five groups in the top 10 rows, as shown in Supplementary Material 4. The above regression equation indicated that the DBIL, LDL, GGT, ALP, GPT, prealbumin, HDL, and triglyceride levels were correlated positively with the MDD diagnosis, while the LDH, UA, TSH, FT3, IDBIL, WBC, FBG, and FT4 levels were correlated negatively (*p*-values < 0.05).

## Discussion

Many potential differentiators between the mood disorders were noticed in stage 1. After that, we started a more in-depth correlation analysis. The levels of four sex steroids (FSH, LH, estradiol, and progesterone), which fluctuate with menstrual cycles, were not compared between females. In consideration of the fluctuation of sex steroids in women's menstrual cycles, we directly put them into stage 2 for further investigation.

A total of five ROCs showed good diagnostic accuracy of these models, where the AUCs were 0.772–0.848. Relatively speaking, the prediction model of women at luteal phase had the highest accuracy, while at follicular phase, it showed the lowest. A relevant study found that severe premenstrual (luteal phase) syndrome is frequently confused with BD (28), and the problem would be well-explained by our database if predicted a few days before menstruation. The AUC of the male model could be regarded as the baseline data, which is similar to the female model at the baseline follicular phase (0.772 vs. 0.777) (34). Therefore, the menstrual cycle may play an important role in distinguishing between the diseases for female patients (13).

Oxidative stress plays potential roles in follicular development, resumption of oocyte meiosis, and steroidogenesis (35–39). Chronic psychosocial stress may affect ovarian function. The role of oxidative stress in medications induces reproductive toxicity as well. Usual menstrual cycle lengths are about 27–29 days in which the follicular and luteal phases take fifty-fifty (40). However, after being grouped by progesterone level and taking out the debatable cases with uncertainty of menstrual phase when we called pre- or post-ovulatory phase (*n* = 112 + 84), we found the rate of female patients with BD in the luteal stage was higher than that of MDD (29.2 vs. 24.7%). The predictive impact of progesterone levels suggests that ovarian follicle development may be suppressed in mood disorders. Compared to each other, follicular maturation in BD is more close to normality than in MDD (about 10% of normal females with no LH surge in the real world) (41). Otherwise, it can be thought that patients with MDD were about 3 years older than patients with BD, affecting the menstrual cycle. Although menopausal women are more likely to have immature follicles, the hormone screening in this study excluded most patients with ovarian function failure (FSH > 25.8 IU/L). We also noticed that even when all disputable cases were added together in the luteal phase, the rate would have been still far from 50%. The progesterone level suggests that ovarian follicle development may be suppressed under psychological and mood disturbances (42, 43). Mood disorders, both in the bipolar and unipolar spectrum, may be associated with decreased fertility rates. We predict patients with MDD may have even poorer potential fecundability. As external interferences on the mature and release of ovarian follicles are irreversible after a LH surge, this difference may explain the luteal model's best diagnostic accuracy. Another possible reason is that these selected patients might not have taken medications before hospitalization, thereby avoiding the medication treatment effect on reproductive function (43). On the contrary, long-term valproate therapy is associated with the development of menstrual disturbances and alterations in the reproductive endocrine system (44) while there has been no evidence of reproductive toxicity in female patients on antidepressant agents (38). As it is not ethical to study the side effects of antidepressants on the reproductive endocrine system in humans, we can only observe them in some inconsecutive reports. Our result is likely to be credible.

The AUC of the periovulation model (transitional model) fell within a place between the two categories above. Indeed, periovulation is not a very precise title as the definition of the ovulation phase is 5 days of preovulation and 4 days of postovulation requiring B-ultrasonography for prospective determination of ovulation (45). Our settings were prior to avoid overlapping of progesterone levels (follicular phase 0.181–2.84 nmol/L vs. luteal phase 5.82–75.9 nmol/L). The ovulation day varies considerably for any given menstrual cycle length (41). To ensure the accuracy of clinical data, we narrowed the reference range of the transitional model from the maximum progesterone level in the follicular phase to the minimum in the luteal phase, which meant more accurate cases were included in the periovulation phase (2.84–5.82 nmol/L) than the expanded reference range (0.385–38.1 nmol/L). The settings are closer to the real conditions.

The difference in the indexes of the hematopoietic system, immuno-inflammatory factors, liver enzymes, glucolipid metabolism, and thyroid and gonadal hormones between BD and MDD will be further discussed. Many previous studies have investigated these biomarker discriminations of BD vs. MDD.

In the hematopoietic system, we found that all of the five indexes had differences between BD and MDD except for RBC and hemoglobin differences, which were found in female patients only. Current evidences show that increased inflammatory cells (both neutrophils and platelets) play an important role in the pathophysiology of BD-mania and the euthymic state (22, 46). A significantly higher percentage of patients with BD have an abnormal (too low or too high) number of platelets compared to unipolar depression, while MDD does not differ in platelet level. The platelets of patients who have MDD with psychotic features are higher than those of patients with other types of depression (47). The differences in RBC and hemoglobin in female patients may result from the rate of follicle maturation related to menstrual bleeding (48).

Four immuno-inflammatory factors are potential indicators [CRP (18), ESR (31), UA (26), and prealbumin (19)] as each of their concentrations are increased in mood disorders. We call them immuno-inflammatory factors because they do not belong to either of the body's organs and systems.

All of those indicators were statistically significant (*p*-values < 0.05), except the ESR in male patients. Various evidences show that CRP (16) and UA (15) levels in BD are increased compared to MDD, while ESR levels are elevated in MDD (30). However, there was no evidence of the usefulness of ESR as an indicator of BD, which limits its predictive usefulness. Although female patients showed more susceptibility to oxidative stress than male patients, these gender-based differences did not seem to provide a biochemical basis for the epidemiological differences (49). Purinergic signaling is involved in the physiology of neurotransmission and neuromodulation (50). MDD might have reduced levels of antioxidant UA (51) while increased levels of antioxidant UA mean accelerated purinergic transformation and/or decreased adenosinergic transmission in BD (52). The purinergic system could prove a promising path for the search of biomarkers in BD.

We grouped liver function and glucolipidmetabolism together as hepatic function is closely related to the cell's metabolism of sugar, fat, and protein under the condition of oxidative stress (20). DBIL, IDBIL, GPT, GOT, and LDH, the commonly used liver function indexes, displayed a series of group differences. Liver oxidative injuries were found in MDD (32) but not in BD. We corroborated the more severe liver–brain interactions in BD under the condition that hepatotoxicity was proved to be unrelated to adverse hepatic events during maintenance mood stabilizers (53). In the previous biochemistry, bilirubin had been regarded as a useless metabolite of heme, and its abnormal rise was only used as a laboratory reference for the diagnosis of jaundice. After its potential antioxidant activity was discovered, we had a more comprehensive understanding of the antioxidative effects of bilirubin (54). It is currently believed that bilirubin is a natural endogenous antioxidant with strong antioxidant activity and mediates oxidative stress (55). We posit that BD may have more severe oxidative stress injury than MDD. Antioxygenation consumes endogenous bilirubin, leading to a decrease of DBIL (water-soluble bilirubin) and a compensatory increase of IDBIL (lipid-soluble bilirubin) by approaches of cellular homeostasis in patients with BD. Antioxidant levels may explicate different changes in bilirubin levels between groups. Metabolic components are significantly associated with BD and MDD in a current depressive episode (25), and impaired glucose metabolism presents a higher ratio of manic/hypomanic than depressive episodes (23). For both sexes, possibly due to the impact on energy metabolism of mood disorders, the FBG level was higher while the triglyceride level was lower when comparing BD to MDD. After the former studies showing liver–brain interactions due to illness vs. health (56), we further figured out some different metabolic characteristics within mood disorders.

We then measured the HPT axis overall and sex steroids subgrouped by sex, especially in women. We found that the TSH, FT4, TT3, and FT3 secretion in HPT axis differed between groups, where the TSH secretion differed only in the male subgroup. We classified by sex in prolactin and testosterone because they are seldom influenced by LH/FSH cycles, and both of them still showed statistical significance in the subgroups. Decreased testosterone secretion was more common in MDD than BD. The investigation of the hypothalamic-pituitary-gonad (HPG) axis yielded that LH and estradiol secretion differed in the male subgroups. Dysfunction of HPT and HPA axes may lead to the different pathophysiology of mood disorders (17, 24, 27).

Prealbumin, also known as transthyretin, is a thyroid hormone-binding protein synthesized by choroid plexus and secreted into cerebrospinal fluid. We therefore put the prealbumin and HPT axis together to discuss. Low levels of prealbumin were identified in BD (21) and MDD (29) in previous studies. The low cerebrospinal fluid prealbumin levels were replicated in depression and bridge the gap between thyroid axis dysfunction and suicidal behavior in MDD (β= −0.58, *p* <0.05) (17, 57, 58). We found that the prealbumin level in BD was even lower than in MDD, indicating a more severe HPT dysfunction in BD. On the contrary, the incidences of low thyroid hormone secretion are significantly greater in MDD than in BD. The HPT dysfunction was replicated by our study, especially FT3 and FT4 as the markers for prediction. We found that FT3 and FT4 in males and FT4 in females at follicular phase may be responsible for the identification of the illnesses.

## Conclusion

We unified the criteria for fixed entry and removal of the regression model, and therefore, the risk factors of each prediction model were not the same. Even so, we could see that the correlations of qualitative analysis of the most robust variables were absolutely consistent across groups.

We used the training sets to predict the data of the independent external test sets and conducted external verification of the models. The effectiveness of the result indicates that our model has fairly good extrapolation application capabilities. The prediction models have good internal and external calibration capability *via* the goodness-of-fit test. Moreover, the diagnostic effect of the model in the test sets is equivalent to that of the training sets, indicating that the model has good applicability (generally the AUC is in the range of 0.70–0.80, and our models fall within this range). The difference may result from regional disparity in diagnosis.

## Limitations

There are some limitations. First, we recorded a patient's blood test when the first definitive diagnosis was given before a standardized treatment procedure was started. However, we could not guarantee every patient was medicine-naïve even on the first hospitalization. Since the data lasted for 10 years with no limitation of drug use, almost every mood stabilizer or antidepressant agent could be on the list of relevant confounding factors. Additionally, medication treatment was reported to have adverse endocrine, hepatic, and metabolic events (39, 53). Although the short-term impacts of medication, tobacco use (59), and alcohol consumption (60) can be eliminated by a set of operating procedures, the long-term impacts of these confounders on peripheral biomarkers were not confirmed in this study. Moreover, we only obtained the diagnosis codes rather than comprehensive quantitative assessment by clinical scales or subdivided the specific clinical symptoms described in a psychiatric interview, even though the three-level ward round was rigorously operated. The analysis of big data is still ongoing, and we are trying to export meaningful clinical data in the future.

## Data Availability Statement

The data that support the findings of this study are available from SMHC. Restrictions apply to the availability of these data, which were used under license for this study. Further enquiries can be directed to the corresponding author.

## Ethics Statement

The studies involving human participants were reviewed and approved by Ethical approval 2019-15R in Shanghai Mental Health Center (SMHC). Permission to take informed consent is formally waived by the approving committee.

## Author Contributions

YZ and ZN designed the study, collected, and analyzed the data. YZ and HJ wrote the first draft of the manuscript. HJ, HL, XW, LY, and JC contributed to data collection and statistical analysis. ZW and JC discussed and commented on the manuscript. YF reviewed and edited the manuscript. All authors read and approved the manuscript.

## Funding

This study was supported by the National Key R&D Program of China (2016YFC1307100), the National Natural Science Foundation of China (91232719 and 81930033), the Scientific Research Project of Hongkou District Health Commission (2101-03), Shanghai Key Medicine Specialties Program (ZK2019A06), Shanghai Clinical Research Center for Mental Health (SCRC-MH, 19MC1911100), Shanghai Mental Health Center Clinical Research Center Special Project for Big Data Analysis (CRC2018DSJ01-1), the Scientific Research Project of Shanghai Municipal Health Commission (202040318), the Key Project of Clinical Research Center of Shanghai Mental Health Center (CRC2018ZD02), and also supported by the Innovative Research Team of High-level Local Universities in Shanghai.

## Conflict of Interest

The authors declare that the research was conducted in the absence of any commercial or financial relationships that could be construed as a potential conflict of interest.

## Publisher's Note

All claims expressed in this article are solely those of the authors and do not necessarily represent those of their affiliated organizations, or those of the publisher, the editors and the reviewers. Any product that may be evaluated in this article, or claim that may be made by its manufacturer, is not guaranteed or endorsed by the publisher.

## Supplementary Material

Supplementary Material 1 Details for the way of data extraction.

Supplementary Material 2 The 35 indexes and their normal ranges.

Supplementary Material 3 The results of no multicollinearity between the 25 remaining independent variables of the gender subgroups.

Supplementary Material 4 These predictors yielding completely consistent performance if more than one predictor was marked among the five groups at the top 10 rows.

## Abbreviations

BD: bipolar disorder
MDD: major depressive disorder
DSM: *Diagnostic and Statistical Manual of Mental Disorders*
ICD: International Classification of Diseases
SMHC: Shanghai Mental Health Center
HSPH: Hangzhou Seventh People's Hospital
HIS: the Hospital Information System
ECLIA: electrochemical luminescence immunoassay
WBC: white blood cell
ESR: erythrocyte sedimentation rate
CRP: C-reactive protein
RBC: red blood cell
UA: uric acid
TBIL: total bilirubin
DBIL: direct bilirubin
IDBIL: indirect bilirubin
GPT: glutamic-pyruvic transaminase
GOT: glutamic-oxalacetic transaminase
LDH: lactic dehydrogenase
GGT: glutamyltranspeptidase
ALP: alkaline phosphatase
FBG: fasting blood glucose
TCH: total cholesterol
HDL: high-density lipoprotein
LDL: low-density lipoprotein
TSH: thyroid-stimulating hormone
TT4: total thyroxine
FT4: free thyroxine
TT3: total triiodothyronine
FT3: free triiodothyronine
LH: luteinizing hormone
FSH: follicle-stimulating hormone
HPT: hypothalamic-pituitary-thyroid
HPG: hypothalamic-pituitary-gonad
ROC: receiver operating characteristic
AUC: the area under the curve.
