# Supplementary material for: Biochemical and Endocrine Parameters for the Discrimination and Calibration of Bipolar Disorder or Major Depressive Disorder
Source: Front Psychiatry. 2022 Jun 20;13:875141. doi: 10.3389/fpsyt.2022.875141 (PMC9251015; doi:10.3389/fpsyt.2022.875141)
Supplement: Supplementary Material 1 — Details for the way of data extraction. [file Data_Sheet_1.pdf]

Supplementary Material 1

The search engine makes up a clinical professional corpus and establishes a corpus for clinical medical record segmentation. Through the technology of Maximum Word Frequency Segment Algorithm, we extracted medical information from the metadata, including diagnoses, medical examinations, operations, medical records, medications, and other heterogeneous data. Using trigger word and core word and trigger word frequency, we realize the fast search for multi-condition related retrieval across data domains and heterogeneous data. At the same time, it provides us advanced search, historical search for expression record, search expression saving, search result export to database of disease bank, and search result export to local terminal. The most important feature of this system is the merging of heterogeneous databases.

Supplementary Material 2

The clinical biochemical data of interest were 35 indexes at admission regarding the hematopoietic system, immune-inflammatory indexes, liver function, glucolipidmetabolism, thyroid function and sex hormones.

This includes: white blood cell (WBC), neutrophil, erythrocyte sedimentation rate (ESR), C-reactive protein (CRP), hemoglobin, red blood cell (RBC), platelet, uric acid (UA), albumin, globulin, prealbumin (transthyretin), total bilirubin (TBIL), direct bilirubin (DBIL), indirect bilirubin (IDBIL), glutamic-pyruvic transaminase (GPT), glutamic-oxalacetic transaminase (GOT), lactic dehydrogenase (LDH), glutamyltranspeptidase (GGT), alkaline phosphatase (ALP), fasting blood glucose (FBG), total cholesterol (TCH), triglyceride, high-density lipoprotein (HDL), low-density lipoprotein (LDL), thyroid-stimulating hormone (TSH), total thyroxine (TT4), free thyroxine (FT4), total triiodothyronine (TT3), free triiodothyronine (FT3), luteinizing hormone (LH), follicle-stimulating hormone (FSH), testosterone, estradiol, progesterone and prolactin.

We collected fasting venous blood between 7:00 a.m. and 8:00 a.m. by a set of standard operating procedures. The inpatients had neither tobacco use nor alcohol consumption at least 18 hours before the blood specimen collection. An electrochemical luminescence immunoassay (ECLIA) was performed using the Roche Cobas e601 automatic electrochemiluminescence immunoassay system, provided by SMHC. After the blood test, clinical medication and dosage adjustment would be arranged by a doctor-in-charge starting at 8 a.m. per day.

To identify these outcomes, we used the reference intervals for common clinical immunology tests (blood cell analysis etc) for comparison, according to the People's Republic of China health industry standards (Chinese Edition WS/T 343-2011/ 404.1-2012/ 404.2-2012/ 405-2012/ 404.7-2015/ 560-2017/ 404.4-2018/ 404.9-2018)--www.nhc.gov.cn/.

The normal range and the unit of measurement of these indexes were listed as follows:

Normal ranges	Total	Male	Female
Hematopoietic system			
WBC(10^9/L)	(3.5,9.5)	(3.5,9.5)	(3.5,9.5)
Neutrophil(10^9/L)	(1.8,6.3)	(1.8,6.3)	(1.8,6.3)
RBC(10^{12}/L)	/	(4.3,5.8)	(3.8,5.1)
Hemoglobin(g/L)	/	(130,175)	(115,150)
Platelet(10^9/L)	(125,350)	(125,350)	(125,350)
Immunoinflammatory indexes			
ESR(mm/h)	/	(/,15)	(/,20)
CRP (mg/L)	(/, 6.0)	(/,6.0)	(/,6.0)
UA(μmol/L)	/	(/,420)	(/,360)
Prealbumin(mg/L)	/	(200,430)	(180,350)
Liver function			
Albumin (g/L)	(40,55)	(40,55)	(40,55)
Globulin (g/L)	(20,40)	(20,40)	(20,40)
TBIL(μmol/L)	(/, 23)	(/,26)	(/,21)
DBIL(μmol/L)	(/,8)	(/,8)	(/,8)
IDBIL(μmol/L)	(/,15)	(/,15)	(/,15)
GPT(U/L)	/	(9,50)	(7,40)
GOT(U/L)	/	(15,40)	(13,35)
LDH(U/L)	(120,250)	(120,250)	(120,250)
GGT(U/L)	/	(10,60)	(7,45)
ALP(U/L)	/	(45,125)	(35,100)
Glucolipidmetabolism			
FBG(mmol/L)	(3.89,6.10)	(3.89,6.10)	(3.89,6.10)
TCH(mmol/L)	(2.9,6.0)	(2.9,6.0)	(2.9,6.0)
Triglyceride(mmol/L)	(0.45,1.69)	(0.45,1.69)	(0.45,1.69)
HDL (mmol/L)	/	(1.04,1.66)	(1.10,1.74)

LDL (mmol/L)	(/,3.12)	(/,3.12)	(/,3.12)
HPT axis			
TSH(mIU/L)	(0.27,4.20)	(0.27,4.20)	(0.27,4.20)
TT4(nmol/L)	(66.00,181.00)	(66.00,181.00)	(66.00,181.00)
FT4(pmol/L)	(12.00,22.00)	(12.00,22.00)	(12.00,22.00)
TT3(nmol/L)	(1.30,3.10)	(1.30,3.10)	(1.30,3.10)
FT3(pmol/L)	(3.10,6.80)	(3.10,6.80)	(3.10,6.80)
HPG axis			
Testosterone(nmol/L)	/	(14.0,25.4)	(0.29,1.67)
Prolactin(mIU/L)	/	(86,324)	(102,496)
LH(IU/L)	/	(1.7,8.6)	Omission
FSH(IU/L)	/	(1.5,12.4)	Omission
Estradiol(pmol/L)	/	(94.8,223)	Omission
Progesterone(nmol/L)	/	(0.32,2.57)	Omission

Supplementary Material 3

We removed these parameters according to the following seven principles:

1. less clinical information (for e.g., neutrophil than WBC);
2. familiar clinical significance but smaller sample size (for e.g., CRP vs. ESR);
3. more interference factors (for e.g., hemoglobin (cell number*volume) than RBC (cell number only);
4. well-replaced by the subcategory (for e.g., TBIL by DBIL plus IDBIL);
5. similar clinical significance (for e.g., GOT with GPT);
6. less precise (for e.g., TCH than LDL and HDL);
7. lower changeable sensitivity (for e.g., albumin, globulin than prealbumin, TT4 than FT4 and TT3 than FT3).

Before building the regression model, the remaining parameters were set to be excluded due to potential multicollinearity under 0.1 of tolerance and above 10 of variance inflation factor. The results showed no multicollinearity between the 25 remaining independent variables of the gender subgroups. The results showed that there is no multicollinearity between the 25 independent variables of the gender subgroups.

The results of multicollinearity analysis between female and male independent variables were listed as follows：

Independent variables	Collinearity statistics			
	Tolerance		Variance inflation factor	
	Female	Male	Female	Male
WBC	0.703	0.682	1.422	1.467
RBC	0.685	0.565	1.459	1.769
Platelet	0.791	0.787	1.264	1.271
ESR	0.738	0.651	1.356	1.536
UA	0.750	0.699	1.333	1.431
Prealbumin	0.885	0.732	1.131	1.366
DBIL	0.840	0.757	1.191	1.321
IDBIL	0.741	0.726	1.349	1.378
GPT	0.599	0.696	1.668	1.437
LDH	0.691	0.716	1.447	1.397
GGT	0.580	0.620	1.724	1.612
ALP	0.806	0.909	1.241	1.100
FBG	0.813	0.848	1.230	1.179
Triglyceride	0.714	0.694	1.400	1.441
HDL	0.832	0.783	1.202	1.277
LDL	0.871	0.836	1.148	1.197
TSH	0.950	0.921	1.052	1.085
FT4	0.411	0.742	2.435	1.347
FT3	0.456	0.691	2.191	1.446
Testosterone	0.817	0.535	1.224	1.868
Prolactin	0.938	0.838	1.066	1.193
LH	0.718	0.701	1.393	1.427
FSH	0.704	0.731	1.420	1.369
Estradiol	0.681	0.635	1.468	1.574
Progesterone	0.641	0.866	1.559	1.155

Supplementary Material 4

The following table summarized the significant correlations (+/-) in those regression models.

	Models				
Predictors	Male	Female	Follicle	Periovulation	Luteum
DBIL	+	+	+	+	+
LDH	-	-	-	-	-
UA	-	-	-	/	-
TSH	-	-	/	/	-
FT3	-	-	-	/	/
IDBIL	-	-	/	/	-
WBC	/	-	-	/	-
LDL	/	+	+	/	+
FBG	/	-	/	/	-
GGT	/	+	/	/	+
ALP	/	/	+	/	/
FT4	/	-	/	/	/
GPT	/	/	/	+	/
Prealbumin	+	/	/	/	/
HDL	+	/	/	/	/
Triglyceride	/	+	/	/	/
